# 28.3 MINIMAL SELF IN SCHIZOPHRENIA: THE TIME PERSPECTIVE

**DOI:** 10.1093/schbul/sby014.118

**Published:** 2018-04-01

**Authors:** Anne Giersch, Brice Martin, Michel Cermolacce, Nicolas Franck, Patrick Poncelet, Jennifer Coull

**Affiliations:** 1 Centre Hospitalier Universitaire de Strasbourg; 2 Université Lyon; 3 Aix-Marseille Université; 4 INSERM; 5 Aix-Marseille Université, Service Universitaire de Psychiatrie, Hôpital Ste Marguerite

## Abstract

**Background:**

The feeling of being one continuous individual in time is a natural evidence, which seems to be lost for patients with schizophrenia who display ‘minimal’ or ‘bodily’ self disorders. The continuity in time is a property of the ‘minimal’ self and its alteration could disrupt the sense of self. It has long been proposed that patients with schizophrenia experience a breakdown of the experience of time continuity. This proposal relies on the patients’ self-reports and the phenomenological analysis of their verbal descriptions. We will discuss to which extent recent experimental evidence supports this proposal and provides insight on the mechanisms underlying the perturbation of the experience of time continuity

**Methods:**

We used two original experimental approaches to test the link between the sense of self and time disorders in stabilized patients with schizophrenia and controls. The first relies on the parallel measure of time expectation and minimal self disorders, as evaluated with the EASE (phenomenological scale). Time expectation is indexed by the ability to benefit from the passage of time to react to a visual target: expectation increases with time, leading to shorter reaction times. The second approach consists in asking subjects to evaluate their feeling of control when tapping with a stylus on a virtual surface. The feeling of control is a component of agency, i.e. related to the bodily self. It can be altered even when subjects know the action to be their own, and may thus show alterations in the absence of delusions. In order to test the link between the feeling of control and timing, the haptic feedback (tactile and kinesthetic) was manipulated, with perceptible or imperceptible delays.

**Results:**

Both tasks show that patients can expect sensory signals and react to unusual events to some extent: they increase their reaction times after trials with missing targets, and their feeling of control decreases when sensory feedbacks are delayed. However, the patients who feel as not being immersed in the world (EASE) do not benefit from the passage of time, consistent with previous results suggesting that patients have a difficulty to fluently follow the events flow. In the motor task, contrary to controls the patients’ feeling of control drops as soon as there is an imperceptible delay in the haptic feedback, and patients have difficulty to adjust sensory anticipation in case of delayed haptic feedback

**Discussion:**

The results suggest a link between timing and minimal self disorders. The patients are able to expect well-learned sensory signals. However, the patients with minimal self disorders (altered immersion in the world) display time disorders consistent with a breakdown of time continuity. All patients display disrupted time expectation when events become unusual or uncertain. Expecting events in time helps to link events with one another and thus participates to transform a chain of discontinuous events in a continuous flow. Conversely, fragile time expectations may lead to a sense of discontinuity, which could disrupt perceptions and especially the flow of bodily signals, thus contributing to bodily self disorders.

